# Supplementary material for: Novel Driver Strength Index highlights important cancer genes in TCGA PanCanAtlas patients
Source: PeerJ. 2022 Aug 11;10:e13860. doi: 10.7717/peerj.13860 (PMC9375969; doi:10.7717/peerj.13860)
Supplement: Supplemental Information 2 [file peerj-10-13860-s002.zip › COHORTS/genes plots/detailed/2021_8_16_14_9_distribution_events_detailed_34.pdf]

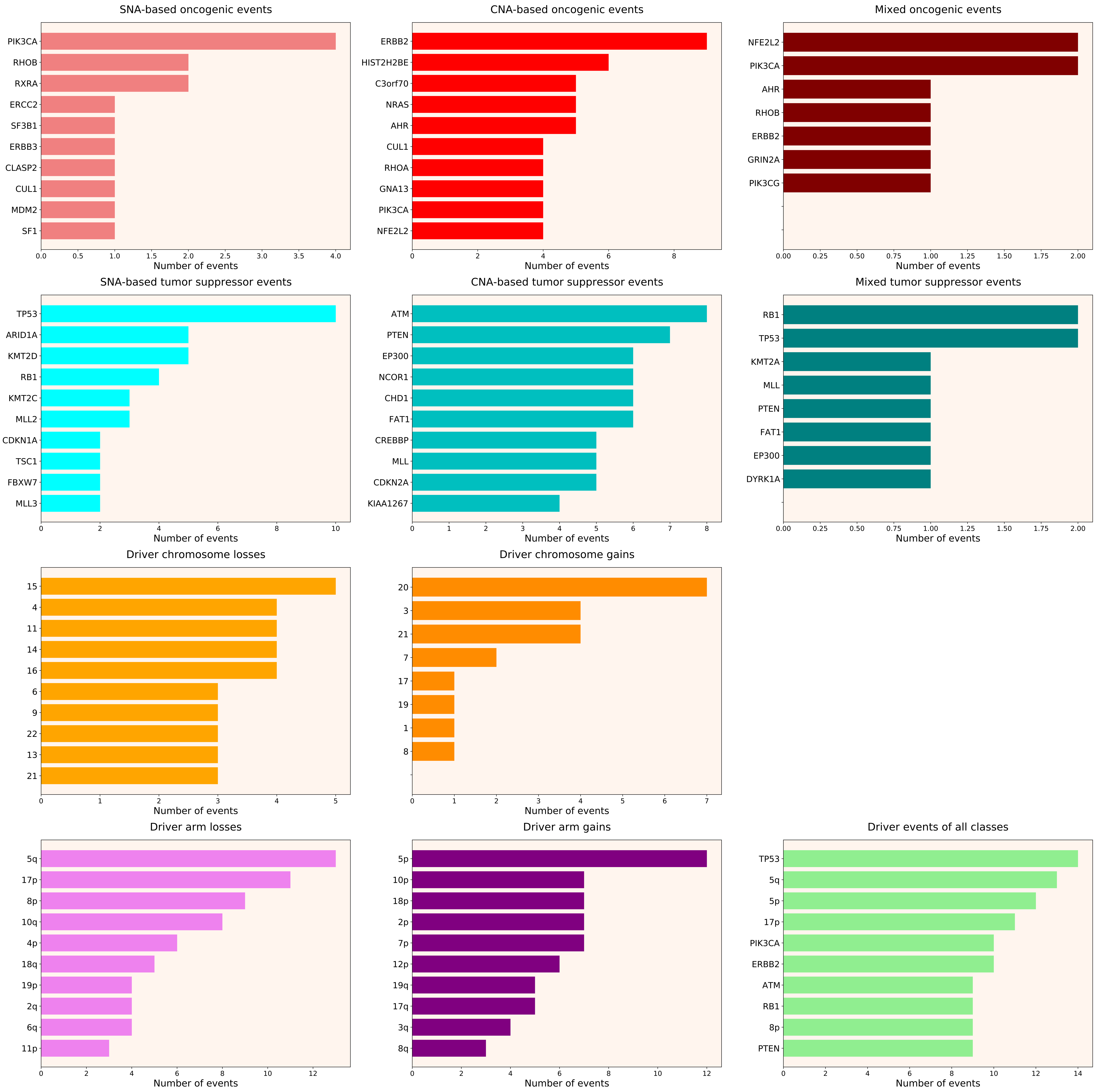
SNA-based oncogenic events
PIK3CA
RHOB
RXRA
ERCC2
SF3B1
ERBB3
CLASP2
CUL1
MDM2
SF1
0.0 0.5 1.0 1.5 2.0 2.5 3.0 3.5 4.0
Number of events
CNA-based oncogenic events
ERBB2
HIST2H2BE
C3orf70
NRAS
AHR
CUL1
RHOA
GNA13
PIK3CA
NFE2L2
0 2 4 6 8
Number of events
Mixed oncogenic events
NFE2L2
PIK3CA
AHR
RHOB
ERBB2
GRIN2A
PIK3CG
0.00 0.25 0.50 0.75 1.00 1.25 1.50 1.75 2.00
Number of events
SNA-based tumor suppressor events
TP53
ARID1A
KMT2D
RB1
KMT2C
MLL2
CDKN1A
TSC1
FBXW7
MLL3
0 2 4 6 8 10
Number of events
CNA-based tumor suppressor events
ATM
PTEN
EP300
NCOR1
CHD1
FAT1
CREBBP
MLL
CDKN2A
KIAA1267
0 1 2 3 4 5 6 7 8
Number of events
Mixed tumor suppressor events
RB1
TP53
KMT2A
MLL
PTEN
FAT1
EP300
DYRK1A
0.00 0.25 0.50 0.75 1.00 1.25 1.50 1.75 2.00
Number of events
Driver chromosome losses
15
4
11
14
16
6
9
22
13
21
0 1 2 3 4 5
Number of events
Driver chromosome gains
20
3
21
7
17
19
1
8
0 1 2 3 4 5 6 7
Number of events
Driver arm losses
5q
17p
8p
10q
4p
18q
19p
2q
6q
11p
0 2 4 6 8 10 12
Number of events
Driver arm gains
5p
10p
18p
2p
7p
12p
19q
17q
3q
8q
0 2 4 6 8 10 12
Number of events
Driver events of all classes
TP53
5q
5p
17p
PIK3CA
ERBB2
ATM
RB1
8p
PTEN
0 2 4 6 8 10 12 14
Number of events
